# Electricity-consumption data reveals the economic impact and industry recovery during the pandemic

**DOI:** 10.1038/s41598-021-98259-3

**Published:** 2021-10-07

**Authors:** Xinlei Wang, Caomingzhe Si, Jinjin Gu, Guolong Liu, Wenxuan Liu, Jing Qiu, Junhua Zhao

**Affiliations:** 1 School of Electrical and Information Engineering, University of Sydney, Sydney, NSW 2006 Australia; 2 Shenzhen Institute of Artificial Intelligence and Robotics for Society (AIRS), Shenzhen, China; 3 School of Science and Engineering, The Chinese University of Hong Kong, Shenzhen, Shenzhen 518116 China

**Keywords:** Energy science and technology, Engineering

## Abstract

Coping with the outbreak of Coronavirus disease 2019 (COVID-19), many countries have implemented public-health measures and movement restrictions to prevent the spread of the virus. However, the strict mobility control also brought about production stagnation and market disruption, resulting in a severe worldwide economic crisis. Quantifying the economic stagnation and predicting post-pandemic recovery are imperative issues. Besides, it is significant to examine how the impact of COVID-19 on economic activities varied with industries. As a reflection of enterprises’ production output, high-frequency electricity-consumption data is an intuitive and effective tool for evaluating the economic impact of COVID-19 on different industries. In this paper, we quantify and compare economic impacts on the electricity consumption of different industries in eastern China. In order to address this problem, we conduct causal analysis using a difference-in-difference (DID) estimation model to analyze the effects of multi-phase public-health measures. Our model employs the electricity-consumption data ranging from 2019 to 2020 of 96 counties in the Eastern China region, which covers three main economic sectors and their 53 sub-sectors. The results indicate that electricity demand of all industries (other than information transfer industry) rebounded after the initial shock, and is back to pre-pandemic trends after easing the control measures at the end of May 2020. Emergency response, the combination of all countermeasures to COVID-19 in a certain period, affected all industries, and the higher level of emergency response with stricter movement control resulted in a greater decrease in electricity consumption and production. The pandemic outbreak has a negative-lag effect on industries, and there is greater resilience in industries that are less dependent on human mobility for economic production and activities.

## Introduction

The outbreak of Coronavirus Disease 2019 (COVID-19) has severely affected the global economy^1^. Many countries have implemented strict mobility control to prevent the spread of the virus, meanwhile resulting in the disrupted economic production activities. As one of the world’s largest economies, China suffered economic stagnation in the first quarter of 2020 with a 6.8% decrease year-over-year in its gross domestic product (GDP)^2^, but then obtained an increase of 3.2% in the second quarter. In efforts of effective epidemic control and gradual reopening of the economy, China became the first country to avert the recession. It is significant to take China as a reference case to analyze the causal relationship between COVID-19 and economic disruption, and COVID-19 and post-pandemic recovery.

As a significant indicator of industrial production and economic activities, the real-time electricity-consumption data can precisely reflect and monitor the real production and operation conditions of enterprises^3–7^. There is bidirectional causality between economic growth and energy consumption in the short run^8–10^. It makes high-frequency electricity consumption data an intuitive and effective tool for not only evaluating the economic impact of COVID-19 but also quantifying the post-pandemic economic recovery. Current studies have revealed noticeable impacts of COVID-19 on the power sector, which analyzed changes in electricity supply & consumption, electricity prices^11–15^, and challenges to grid stability and ancillary services^16,17^. To quantify the impact on electricity consumption during the pandemic, some prior works have reported or estimated the general or static changes in energy demand and related carbon emissions in 2019 and 2020^18–20^. However, there is no detailed analysis model to reveal the causal relationship between factors such as electricity consumption and the specific pandemic-related policies, and post-pandemic economic recovery. Besides, there is no analysis based on a dataset that covering electricity consumption data of all industries. To examine the economic impact of COVID-19 on different industries, some economists have conducted empirical analyses based on the survey results, stock price, or annual change in the financial performance of firms^21–23^. However, these data lack finer data granularity^24^, and most are not at the high-frequency daily level or county level, which are delayed reported information. Although macroeconomic data can be used to evaluate the overall impact, there is still a lack of real-time fine-grained analysis to assess the economic stagnation and recovery. Not only the effect of COVID-19 on economic activity but also the asymmetry of the distribution of economic impact among industries remain unclear. The lack of such fine-grained analysis will lead to inaccuracy and uncertainty in policy formulation, making it difficult to deal with the trade-off between economic development and epidemic control. As a viral pandemic has threatened industries, it is also necessary to examine how the COVID-19 resilience varied with industries to secure business continuity. However, the basis for policy intervention and conceptual frameworks for industry resilience^25^ are far from definitive, and gaps in knowledge remain. Therefore, quantifying the economic impacts due to COVID-19 with high temporal resolution and figuring out the mechanism of COVID-19’s effects on the power sector have become emerging issues.

**Figure 1:**
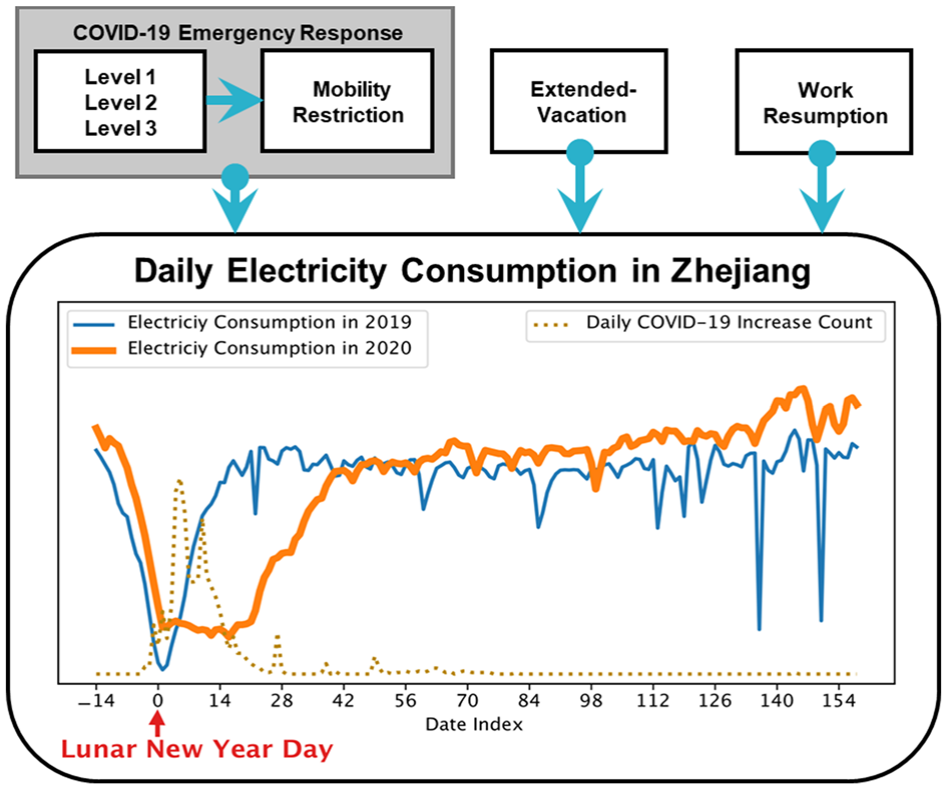
The daily electricity consumption during the COVID-19: the figure compares the actual daily electricity consumption in eastern China in 2020 to itself in the same matched lunar calendar period in 2019. The horizontal axis represents the date index, and day 0 represents the Lunar New Year day (2020: Jan 24th; 2019: Feb 4th). The dashed line is the daily COVID-19 increase cases in eastern China.

In this paper, we aim to answer three questions: (1) how does the COVID-19 affect regional electricity consumption? (2) how does the economic effect of the COVID-19 vary across different industry groups? and (3) how to quantify the resilience and post-pandemic recovery of different industries? A detailed analysis model of how control measures have modified the electricity consumption in eastern China has been performed in this work. We employ a set of difference-in-difference (DID) estimators^26^ to disentangle the causal impacts of these public-health measures on market electricity consumption from other confounding effects such as the seasonal Spring Festival. A large-scale electricity consumption dataset is collected to support the aforementioned analysis, which records the daily electricity consumption at the county level, covers a large population, and involves 53 types of industries in eastern China. In analyzing the mechanism of COVID-19 affecting electricity consumption, our work considers different public-health measures as exogenous variables. The DID estimation model can estimate the causal impacts of a specific intervention while mitigating the effects of unobserved extraneous factors and selection bias. Details of the sample selection and DID modeling can be found in the Methods. Our model takes the 2019 data as an appropriate counterfactual to estimate a causal effect by removing the bias of constant difference between the 2020 and 2019 data. The coefficient of a policy dummy variable in the model measures the average treatment effect on the treated (ATT). Figure 1 compares the actual electricity consumption curves of eastern China in 2020 and 2019. Differences in two groups of regional economic activities (with or without COVID-19) illustrate the economic impact during the pandemic, the extent of which depends on pandemic-related policies and their implementation time, including mobility control due to COVID-19 emergency response, vacation extension of Spring Festival, and work resumption in February 2020.

To the best of our knowledge, this paper is the first to examine the causal impacts of COVID-19 and relevant control measures on economic activities based on the electricity consumption data. We are among the first to collect a large-scale electricity consumption dataset covering such many industries during the pandemic. We propose a detailed analysis model to quantify the resilience and post-pandemic recovery of different industries in response to COVID-19, which should be specified for future economic support measures in response to the public health crisis. This paper has produced three key findings. First, this study provides empirical evidence for the negative impacts on electricity consumption during the pandemic. The estimation results show that all levels of emergency response, the combination of pandemic countermeasures in a certain period, affected electricity consumption of almost all industries other than the information transfer. Second, we also explored the post-pandemic recovery among different industry groups. Until May 2020, economic activities of all sub-sectors started to bounce back from the virus slump, and the removal of control measures even stimulus the increase in several industries compared to the pre-pandemic trend, such as education, building construction, retail, agriculture, etc. Third, we compared the multi-period pandemic impacts on industries and found that sub-sectors that heavily rely more on in-person interaction for economic activities suffer economic stagnation longer. There is greater resilience in industries that are less dependent on human mobility.

The paper is structured as follows. In Section Results, we (1) define 4 main phases to support multi-period analysis, (2) quantify the impact of the pandemic on regional electricity consumption and 53 subsector’s electricity consumption, (3) and finally, we discuss the resilience and post-pandemic recovery of different industries. In Section Methodology, we describe the dataset and use various DID estimations to evaluate the pandemic effect on electricity consumption. Section Discussion concludes.

## Results

**Figure 2:**
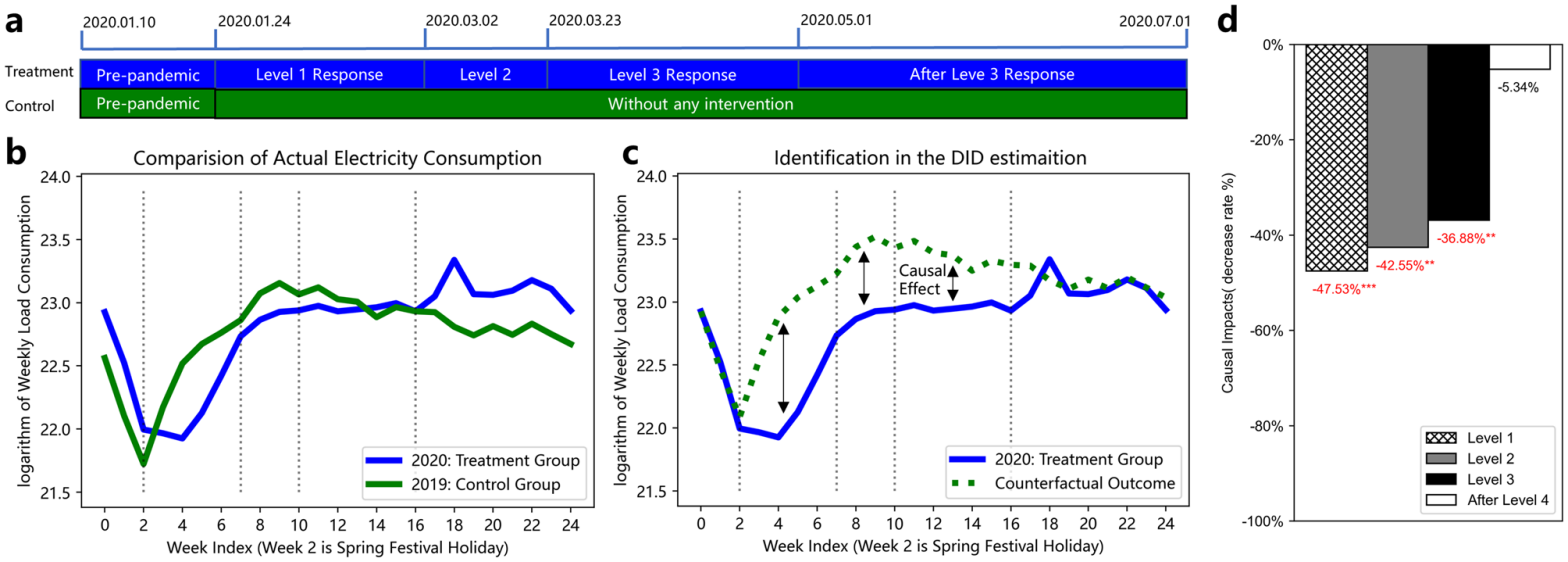
(**a**), the timeline of the four main phases and their corresponding interventions during the pandemic. (**b**) The comparison of the actual weekly electricity consumption in 2020 to the same matched lunar calendar period in 2019. (**c**) The difference between actual weekly electricity consumption in 2020 and the identical counterfactual electricity consumption without any intervention. The average difference between the two groups is estimated as the causal effect. (**d**) The estimation results of causal effects on total electricity consumption in Zhejiang. Each bar represents one level of emergency response. *** means significant at the 1 percent level. ** means significant at the 5 percent level.

The data collected from Zhejiang province are used in this study, including 96 counties and covering 57 million people. It records the daily electricity consumption from January 1st, 2019 to July 1st, 2020. The involved industries are categorized into three main economic sectors and 53 sub-sectors. We define four main phases to support multi-period analysis, as illustrated in Fig. 2a. These phases are divided by iconic policies, which can measure the main stages of the evolution of the pandemic. The first phase starts on January 24th, ends on March 1st, and corresponds to the level 1 emergency response announced^27^. During this period, the strictest movement restriction was implemented at first and the lunar New Year holiday was extended to four weeks. After February 9th, the quarantine policy was gradually liberalized and industrial production began to reopen. The second phase starts from the downgrading of the emergency response level to level 2 on March 2nd and ends on March 22nd. In the second phase, closed management was implemented in communities, and people were encouraged to work from home to ensure normal work and production. The third phase followed and we take the Chinese Labour Day on May 1st as the end time of the third phase, since at that time mobility in Zhejiang has already returned to the pre-pandemic levels^28,29^. The rest period is defined as the fourth phase.

### Causal effect estimation of health measures intervention

Given the definition of these four phases, we use DID model to estimate the causal effect of these control measures, where we consider the multi-period effects in the temporal domain. Figure 2b compares the actual electricity consumption in 2020 to itself in the same matched lunar calendar period in 2019 and takes the 2020 data as the treatment group and the 2019 data as the control group. Figure 2c presents an identical counterfactual electricity consumption without any intervention, which is marked by the green dashed line. The causal effect is the difference between the treatment group and counterfactual outcome, highlighted by arrows. Here, we use weekly statistical data to fulfill the parallel trend assumption^30^, ensuring that the difference between the treatment and control group is constant over time in the absence of public health measures caused by the pandemic in Figure 7. The model takes the 2019 data as an appropriate counterfactual after deducing the constant difference between these two groups. The causal effect is estimated after removing biases in post-intervention period comparisons between two groups, as well as biases from comparisons over time in the treatment group. Differences in two groups of regional economic activities (with or without COVID-19) illustrate the economic impact during the pandemic, the extent of which depends on pandemic-related policies and their implementation time, including mobility control, extended vacation, and work resumption.

We report the results from the DID model and analyze the causal effect on total electricity consumption in Figure 2d. The DID estimation results indicate that the level 1 emergency response had the most severe impact. This corresponds to the impact of the most strict movement restriction and a 24/7 agency-wide effort^31^. Higher emergency response level measures bring about greater reductions in electricity consumption. As shown in Fig. 2d, the emergency response of level 1,level 2, level 3 led to a 47.53%, 42.55%, and 36.88% decrease in regional electricity consumption, respectively. Besides, results also show that the regional electricity consumption gradually returned to the pre-pandemic levels after relaxing the movement restriction during the fourth phase. It is worth noting that the traditional method of directly calculating the year-on-year reduction rate ignores the permanent difference between the 2020 and 2019 data, and underestimates the causal impact of these pandemic-related measures. Although the actual electricity consumption in Zhejiang has already reached the pre-pandemic level during the third phase as shown in Fig. 2b, after deducting the permanent difference of group-specific means, the causal effect estimated by the remaining differences between two groups is still significant during the third phase, shown in Fig. 2c.

**Figure 3:**
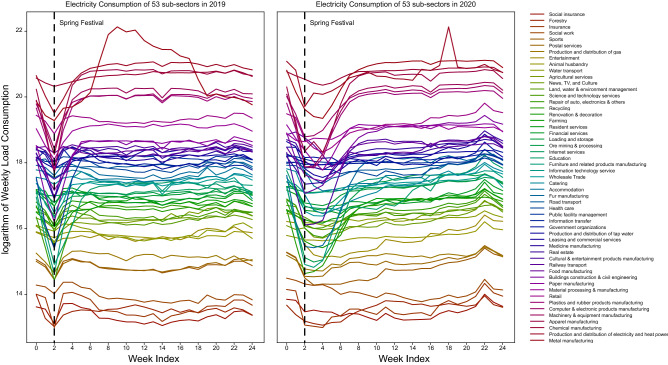
Weekly electricity consumption of 53 sub-sectors in 2019 and 2020: each line represents the logarithm of the electricity consumption of one sub-sector.The vertical dashed line represents the week of the Spring Festival Holiday.

### Responses of different sub-sectors to control measures

**Figure 4:**
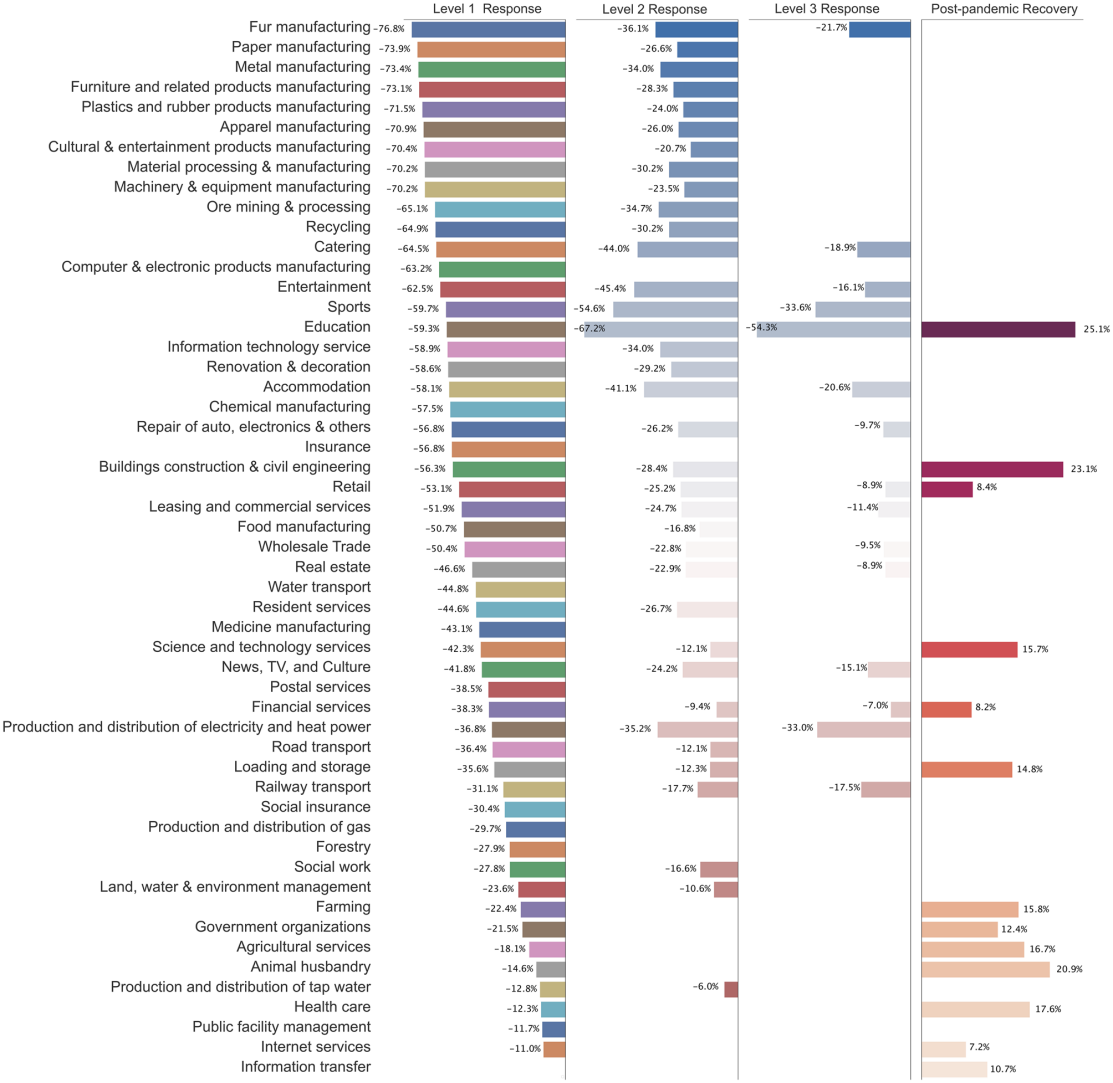
Multi-period causal impacts on electricity consumption of 53 sub-sectors.

**Figure 5:**
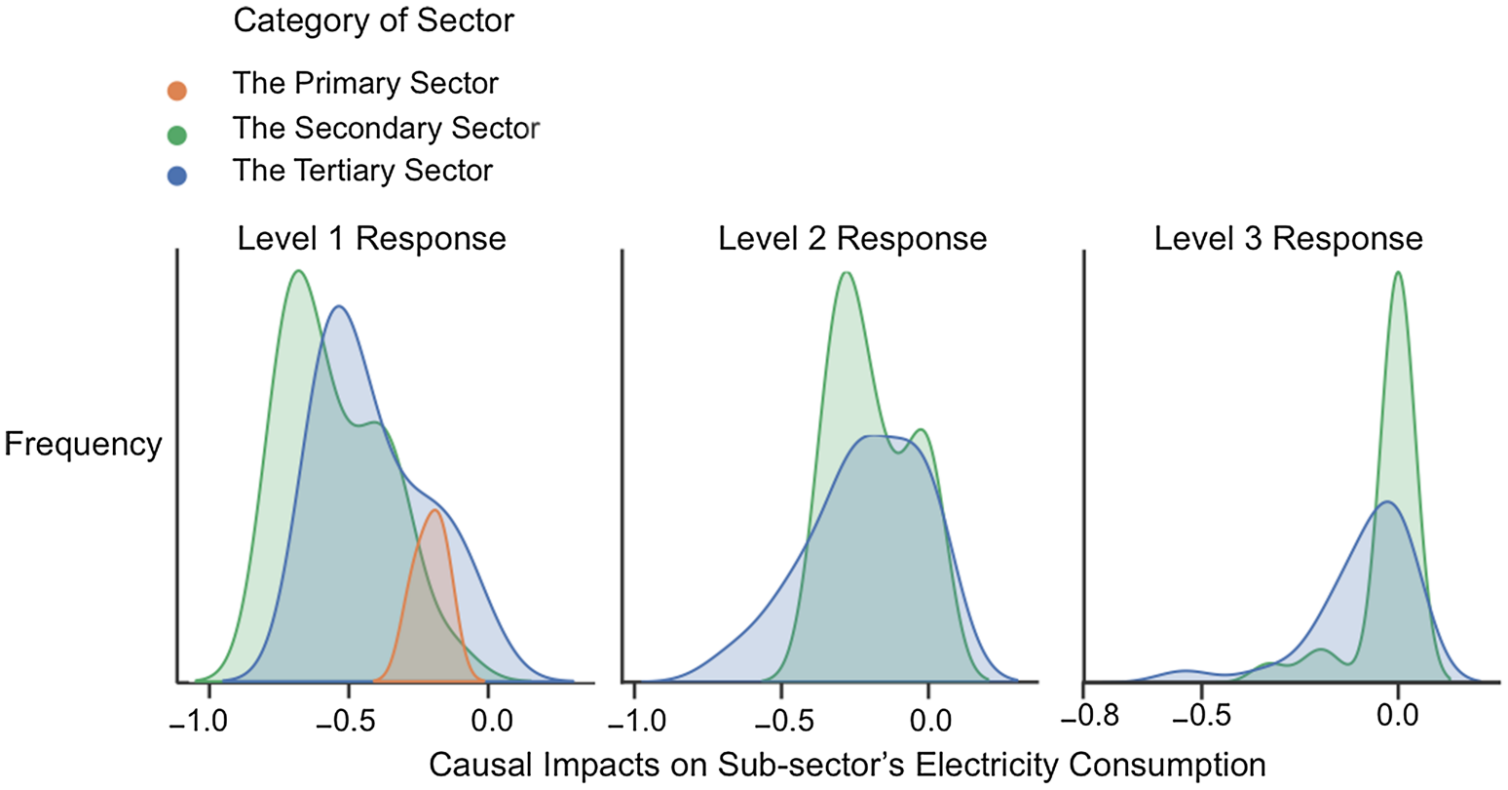
Distribution of causal impacts on three main economic sectors: kernel density estimate (KDE) plot represents the DID estimation results using continuous probability density curves. The data covers the multi-period causal effects of 53 sub-sectors which are classed as three main economic sectors.

Comparing the electricity consumption of all 53 sub-sectors in 2020 and 2019 in Fig. 3, COVID-19 has caused a long-term stagnation in all industries. To quantify and compare the economic stagnation and the recovery rate of different industries, we construct DID estimation for 53 sub-sectors on their county-level electricity consumption data. Figure 4 illustrates the causal effects and compares post-pandemic recovery rate for sub-sectors during the four phases. The result indicates that a higher level of emergency response affects a wider range of industries with greater reductions in electricity consumption. As shown in Fig. 4, level 1 emergency response severely impacts the electricity consumption of all sub-sectors (other than information transfer). Among all, manufacturing is worst influenced during the first phase, in which factories are forced to postpone their production due to extended-vacation policy and strict quarantine policies. With supply chain bottlenecks, the COVID-19 pandemic ushered in challenges to industrial manufacturers, especially those that depend on workers whose jobs cannot be carried out remotely. Besides, the service industry, such as “catering” and “accommodation”, also experienced a serious crisis. For service organizations, customer panic about the COVID-19 and strict control of social distancing may be the main cause of the disruption of their normal operation as well as the customer loss. It requires service organizations to have the flexibility for the duration of both the current public-health interventions and the eventual economic recovery.

After downgrading emergency response to Level 2, 68% of sub-sectors were still suffering stagnation due to the control measures. Several industries have returned to the pre-pandemic level in the second phase, including sub-sectors of “insurance”, “water transport”, “postal service”, “medicine manufacturing”, etc. It can be explained that Zhejiang gradually removed mobility and activity restrictions and reopened industrial production during the second phase. The government has prioritized essential sectors, specific industries, regions, and population groups based on ongoing risk assessments^32^. Education became the industry mostly influenced by the pandemic during this period since all schools and educational institutions were shut down while students were required to stay at home. Service organizations such as restaurants and retailers which rely on customer footfall have been significantly affected since all firms face supply chain challenges due to traffic restrictions, limitations on cross-provincial procurement, and a lack of delivery capacity^33^.

In the third phase, most businesses and factories have reopened nationwide, but social distancing rules remain in place at the micro-level, and foreign entry remains restricted to contain imported cases. Localized movement restrictions have were re-imposed in new hotspots but have been lifted subsequently. Consequently, 28.4% of sub-sectors were still affected by the control measures, while 71.6% of sub-sectors have already returned from the virus slump. The level 3 emergency response mainly affected sub-sectors in the service sector which rely heavily on in-person interaction, including sub-sectors from catering and accommodation to financial services, rail transport, and entertainment. Physical distancing, reduction of nonessential operations, and limited contact raise fundamental challenges about how the industry can reach customers and meet their expectations. At the same time, most industrial manufacturers were recovering from the impact of the pandemic with normalizing economic activity. We take the Chinese Labour Day on May 1st as the beginning time of the fourth phase, since it was the first peak travel season of the year and at that time mobility in Zhejiang has returned to the pre-pandemic level.

During the fourth phase, economic activities of all sub-sectors started to bounce back from the virus slump, and the removal of control measures even stimulus the increase in the electricity consumption of industries compared to their pre-pandemic production scales. We can divide these industries into three categories: (1) the first category refers to industries increasing the workload to make up for delayed production during the pandemic, such as “education”, “building construction civil engineering”; (2) the second category refers to industries significantly contributing to the pandemic control, such as “health-care”, “science services”, “loading storage”, and “information & internet services”; (3) the third category refers to industries that benefit from post-pandemic “retaliatory rebound” in consumption, such as “retail”, “financial services”, “farming” and “forestry” in agriculture.

Figure 5 shows the distribution of causal effects on sub-sectors in the primary sector, the secondary sector, and the tertiary sector. We categorize all 53 sub-sectors into three main economic sectors. Our results indicate that the secondary and tertiary sectors were the worst affected due to strict control measures. The primary sector (including farming, forestry, animal husbandry, and fisheries) only was affected by the level 1 response. Causal effects on the primary sector at the first phase are mainly affected by the pandemic considering the stagnation of production, disrupted agricultural products supply, and farmer’s employment. Immediate countermeasures of the government stimulus the recovery of the agriculture at the second phase, including resuming production and farmers’ work, and financial supports, .etc.

**Figure 6:**
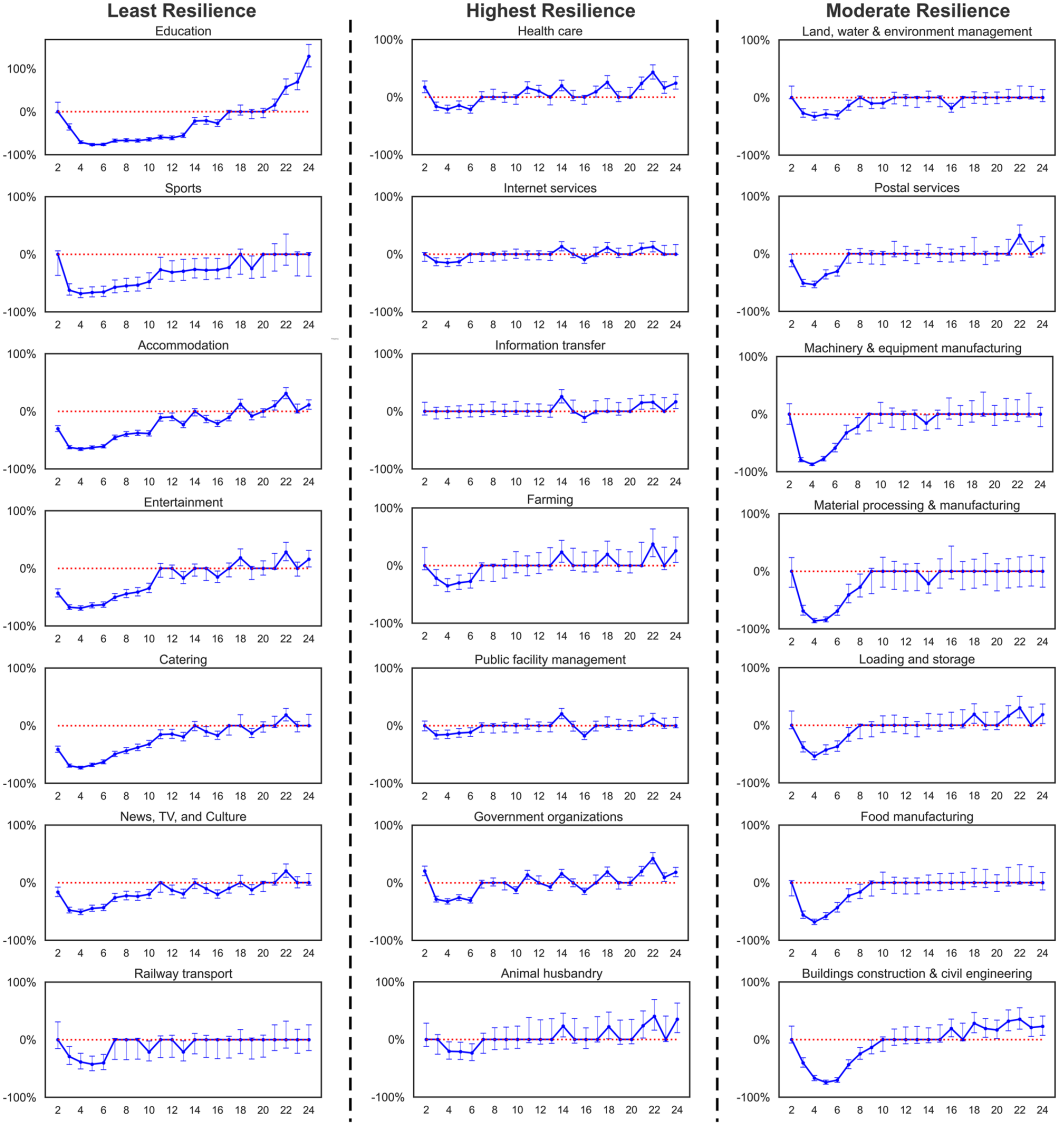
The dynamic causal effect of the COVID-19 outbreak on different industries. The vertical axis represents the reduction rate of electricity consumption due to the COVID-19 outbreak. The horizontal axis represents the week index, the 2nd week is the week of the Spring Festival Holiday as well as the COVID-19 outbreak. The blue line shows the dynamic causal effect of COVID-19 for the specific sub-sector from the 2nd week to the 24th week. The error bar represents the 95% confidence interval. The red dashed line is the baseline representing the situation that there is no pandemic effect.

### Industry resilience to a public health crisis

We define the industry resilience to a health emergency from two aspects: one is how much loss in production it would cause due to the crisis, the other is how long it would require the industry to return to the pre-pandemic trends from the virus slump. Uncertainty surrounding the duration of the pandemic makes it important to examine how a recovery could unfold for the industry, considering the widespread quarantines, the extension of vacation, the extension of school closures, and travel restrictions. We compare the dynamic causal effect of the COVID-19 outbreak on 53 sub-sectors weekly electricity consumption from Jan 24th to Jul 1st in 2020. Figure 6 illustrates the dynamic causal effects of the COVID-19 outbreak or the reduction rate of electricity consumption in each week due to the COVID-19 outbreak. Estimation results show that although all industries were affected due to the pandemic outbreak, the reduction of their electricity consumption, as well as the time required for recovery, are varying with industries with a negative-lag effect covering several weeks. We define the recovery period for one sub-sector as follows: the recovery period starts from the time when the pandemic first brings the negative effect on the sub-sector’s electricity consumption, and it ends until the sub-sector returns to a pre-pandemic level with non-negative effects on its electricity consumption.

The recovery from the pandemic shock for all sub-sectors requires a time of 7 to 15 weeks. In the face of a crisis or economic slowdown, organizations that require less recovery time from the virus slump remained resilient. In Fig. 6 we categorize sub-sectors into three main categories in terms of their resilience to COVID-19, including industries with the least resilience, moderate resilience, and highest resilience. According to the ranking of sub-sectors depending on their post-pandemic recovery, “agriculture”, “internet service”, and “health care” are the most resilient sub-sectors, while “education”, “sports”, and “accommodation” in service are the least resilient. “Manufacturing” with moderate elasticity suffered production interruptions only before the 10th week under the strictest movement control. Once workers and farmers returned to work, these industries soon returned to the pre-pandemic level of electricity consumption.

The pattern of dynamic causal effects of the COVID-19 outbreak can be explained by the relevant control measures in each period. The results show that the impact of COVID-19 on the industry during and after the Spring Festival holiday presents different patterns. At first, “entertainment”, “catering”, and “accommodation” were severely affected due to the outbreak. Conversely, the outbreak resulted in fewer impacts on the secondary sector than the other two economic sectors. In China, the traditional approach is that since most employees have gone home on holiday, the secondary industry usually closes at that time. After the first week of the Spring Festival, China decided to postpone the regular work resumption and extended the holidays from January 24th to February 9th in 2020. Therefore, almost all sub-sectors first experienced the greatest reduction of their electricity consumption from the 3rd week to the 6th week, which is caused by the strict movement control and the extended vacation of the Spring Festival holiday. Started from the 3rd week people were required to stay at home and could not return to work due to the announcement of extending the vacation. Manufacturing then became the most influenced industry since their normal production schedules were delayed under the unexpected and forced shutdown. Therefore, the economic activities of the secondary sector experienced the worst damage during this period. Service organizations were still affected after the vacation due to the pandemic, including “accommodation” and “entertainment”. Especially for education that had the least resilience during the pandemic, the national lockdown of education institutions has caused a major interruption in students’ learning, disruptions in internal assessments, and the cancellation of public assessments for qualifications^34,35^. Returning from the virus slump, more and more industries have resumed working while the government gradually relaxed the control measures. Among all, industries that actively contributed to the anti-epidemic have less damage, and the scale of their economic activities even increased during the pandemic, such as “public facility management”, “healthcare”, “information transfer”.

## Discussion

Our results are in line with the latest studies’ results and public report^19,20,36–39^, showing that electricity demand in China rebounded sharply after the initial shock, and is back to pre-pandemic trends in the 2020 third quarter. Past studies mostly rely on year-on-year change rates to quantify the pandemic effect on electricity consumption. As reported by International Energy Agency (IEA) in 2020^39^, the change rates of electricity demand in commercial and services are -8%, 1%, 6% in the first, second, and third quarter of 2020, while that of industrial activity are respectively -9%, 4%, 6%. After comparing our estimation results with previous studies, we found that the traditional approach of calculating the year-on-year reduction rate without dealing with confounding variables underestimates the negative impact of COVID-19 control measures. For example, our results in Fig. 4 that all industrial sub-sectors (e.g., manufacturing) experienced a decline of over 70% due to level 1 emergency response (from Jan 24th to Mar 1st, 2020), and then decline by over 20% due to level 2 emergency response (from Mar 2nd to Mar 23rd, 2020). The reason why the difference occurs is that the year-on-year reduction rate ignores the permanent difference between the 2020 and 2019 data that may cause by unobserved variables and cannot avoid the omitted variable problem and selection bias problems. In addition to analyzing industrial activity and service industry, our work also contributes to propose a detailed analysis model to quantify the resilience and post-pandemic recovery of 53 sub-sectors in response to COVID-19, covering the primary, secondary, and tertiary sectors.

During the pandemic, the magnitude of the impact on the power sector is determined by consumer and firm behavior in the face of adversity and uncertainty, and public policy responses. This transition results in a significant change in electricity demand levels and patterns. As a reflection of enterprises’ production output, high-frequency electricity consumption data is an intuitive and effective tool for evaluating the economic activities of different industries. In this paper, we propose to examine the multi-period causal effects of the pandemic-related control measures on electricity consumption. A set of DID estimation models are employed to disentangle the COVID-19 effect on market electricity consumption from other confounding effects, including Spring-festival and holiday effect. According to the policy timeline, we define four main phases to support multi-period analysis. We first quantify the effects on regional electricity consumption and then examine effects on different sub-sectors. Among all, the secondary and tertiary sectors are the worst affected, especially for labor-intensive industries. In contrast, although the agricultural sector was affected during the first phase, it had the least impact during the following phases. After gradually returning to work in February 2020, sub-sectors that rely more on human mobility and in-person contacts are more likely to suffer severe economic stagnation, such as education, catering, accommodation, and sports. Conversely, industries that actively contributed to the anti-epidemic have less damage from the pandemic, such as the public administration, social insurance, social organizations, and healthcare. The lag effects of the COVID-19 outbreak show the long-term disruption of economic activities due to the pandemic. Some sub-sectors (*i.e.*, sub-sectors with negative recovery rate in phase 4) require a longer time to return to normal production, while some sub-sectors (*i.e.*, sub-sectors with positive recovery rate in phase 4) have expanded their economic production output compared to the pre-pandemic trend after easing the control measures. It is necessary to formulate policies based on the response and resilience of different sectors under strict public health measures.

Overall, this paper has produced three key findings, and the corresponding suggestions are as follows:

1. *More Precise Quantification of Economic Impacts due to Pandemic* This study provides empirical evidence for the negative impacts on electricity consumption during the pandemic. After comparing our model results with previous studies, we found that the traditional approach of calculating the year-on-year reduction rate without dealing with confounding variables underestimates the negative impact of COVID-19 control measures. Meanwhile, different categories of emergency response have different levels of negative impacts on the electricity consumption of all industries. A higher level of emergency response with stricter movement control resulted in a greater decrease in electricity consumption and a wider range of industries affected. Our model provides a detailed analysis method for the policymakers to precisely quantify the economic impacts of different control measures and rethink the trade-off between restricting the movement of people and stimulating economic recovery. With the normalization of pandemic control, it is of great significance for accurately predicting the corresponding changes in electricity consumption under public health crises.
2. *High-frequency electricity consumption dataset for production recovery evaluation* We also explored the post-pandemic recovery among different industry groups. Until May 2020, economic activities of all sub-sectors started to bounce back from the virus slump, and the removal of control measures even stimulus the increase in several industries such as education, building construction, retail, agriculture, etc. A real-time electricity consumption dataset for different industries can be collected for analyzing the recovery patterns of firms. The post-pandemic stimulus measures should be based on the analysis and drive investment in infrastructure.
3. *Resilience prediction for public health crisis* We compared the multi-period pandemic impacts on industries and found that sub-sectors that heavily rely on in-person interaction for economic activities suffer economic stagnation longer. There is greater resilience in industries that are less dependent on human mobility. Different industries show a different level of resilience to COVID-19 and recovery patterns. The policymakers and firms themselves should learn a lesson to know more about the resilience property of industries before the public health crisis. Therefore, the industries with less resilience should be supported and prepared in advance, such as education, sports, entertainment, accommodation, and railway transport.

## Methods

### Data acquisition

We collected the daily electricity consumption data for 53 sub-sectors in 96 counties in Zhejiang Province, and the data cover the time period from January 1st in 2019 to July 1st in 2020. The sub-sectors, covering the primary sector, the secondary sector, and the tertiary sector, are classified based on the Sectoral Classification System GB/T4754-2011^40^. There are in total 22 main sectors and 53 sub-sectors. Due to the large dynamic range of electricity consumption between different industries, we pre-process the data by taking the logarithm.

### Empirical strategy

We applied the DID method to compute the ATT of the emergency response during the pandemic on the industry’s economic activities based on the electricity consumption data across the Eastern China. DID model is suitable for estimating causal effects of certain policy interventions and policy changes when conditions are satisfied as follows^26^: (1) research designs are based on controlling for confounding variables; (2) using instrumental variables is deemed unsuitable; (3) at the same time, pre-treatment information is available. In the context of the industry’s electricity consumption affected by the pandemic in 2020, empirical designs can adopt the 2019 data as the control group since the distribution of industry groups and trend of the production activities remain almost unchanged during these two years^41^. The pre-treatment information is also contained in our dataset. Meanwhile, there is no available instrumental variable for the analysis of electricity consumption.

There are three common challenges to estimating causal impacts, namely the selection bias, omitted variable bias, and model dependence^42^. Cross-sectional studies have applied the outcome of units in the control group as the counterfactual, which potentially leads to selection bias in such a way that proper randomization may not be achieved. DID method is intended to mitigate the effects of extraneous factors and selection bias, depending on how the control and treatment groups are chosen. Our empirical designs avoid the selection bias by comparing the county-level electricity consumption in eastern China in 2020 to itself in the same matched lunar calendar period in 2019. The second major concern is that the treatment variable becomes endogenous. That is to say, the assignment when the treatment group may be correlated with unobservable variables that also influence the outcome of interest. For instance, the weather and temperature in a region could be an unobservable variable that influences the industry electricity consumption in a region since a rising temperature would increase electricity consumption for heating and cooling. Extreme weather would influence the production activity of local residents. Another major concern is model dependence. The actual relationship between variables may not be consistent with the assumed models. The conditional expectation function could be non-linear and lead to biased estimation.

To address these concerns, we used the DID method controlling the fixed effects to estimate the ATT of the emergency response during the pandemic on the industry electricity consumption. Fixed effects include the time fixed effect and county fixed effect. The parallel trend assumption is tested by introducing the pre-pandemic period indicators. We also adopted Zivot-Andrews structural-break unit-root test^43^ to check whether there is any structural break point. Below we describe each method in detail.

### DID model

Difference in differences (DID) is a statistical technique to mimic an experimental research design using observational study data to estimate the causal effect. It calculates the effect of a treatment (*i.e.*, an explanatory variable or an independent variable) on an outcome (*i.e.*, a response variable or dependent variable) by comparing the average change over time in the outcome variable for the treatment group, compared to the average change over time for the control group. Four groups of objects are used in DID design. They are the group which already received the treatment (post-treatment treated), the group which is the treated prior to their treatment (pre-treatment treated), the group which is the non-treated in the period before the treatment occurs to the treated (pre-treatment nontreated), and the non-treated in the current period (post-treatment nontreated). The idea of this empirical strategy is that if the two treated and the two non-treated groups are subject to the same time trends, and if the treatment has had no effect in the pre-treatment period, then we can use the mean changes of the outcome variables for the non-treated over time and add them to the mean level of the outcome variable for the treated prior to treatment to obtain the mean outcome the treated would have experienced if they had not been subjected to the treatment. The mean outcome is the estimation of the treatment effect.

The basic set-up of DID method requires the time fixed effect, unit fixed effect, treatment dummy variable $Treat^t$ for whether the unit is in a never-treated group, indicator dummy variables (or policy dummy variable) $P^t$ for whether the unit has been treated by time *t*, and finally the error term that is time varying unobservable. The coefficient of $\mathtt {Treat^t}\cdot \mathtt {P^t}$ is the parameter of interest, which is interpreted as the average treatment effect of treated. To compute the ATT of the emergency response during the pandemic on the industry electricity consumption, we employ the same empirical strategy based on DID, and we regress the observed outcome on the treatment variable and a full set of unit- and time-fixed effects. Here, since we collect the county-level electricity data for 53 sub-sectors, each unit in our models is a specific county. Also, there are in total three levels of Emergency response to the pandemic in China, so we set three policy dummies $\mathtt {After^{t}_{1,2,3}}$to figure out the effect of each level and one policy dummy $\mathtt {After^{t}_{4}}$ for the post-pandemic recovery. To test the parallel trends assumption, we introduce an indicator dummy variable $\mathtt {Before^t}$ and if the coefficient of $\mathtt {Treat^t}\cdot \mathtt { Before^t}$ is statistically insignificant, the parallel assumption is satisfied.

**Figure 7:**
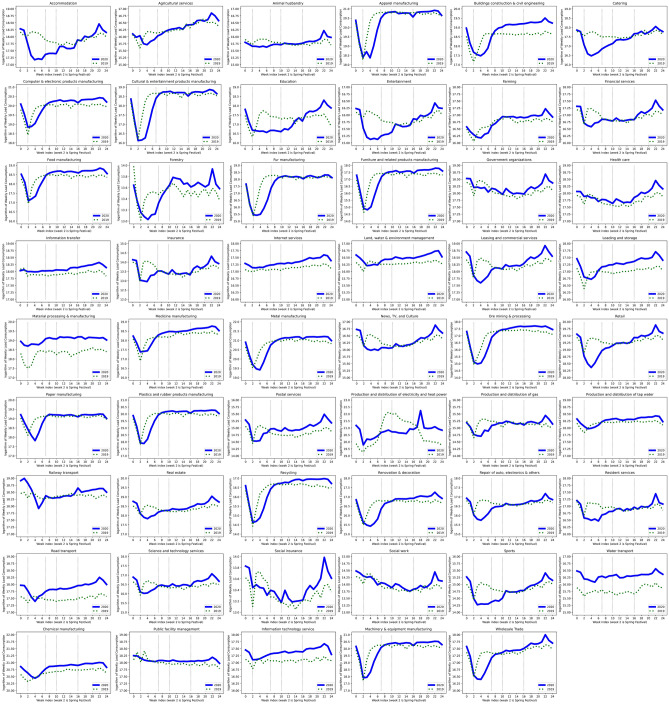
The Comparison of the Weekly Electricity Consumption of 53 Sub-sectors in 2019 and 2020.

In this paper, we use a difference-in-difference specification based on the regression set-up in Eqs. (1), (2), and (3). Repeated cross-section data follow different groups of sectors (*e.g.*, the electricity consumption of sectors in the successive day) that are clustered within the same counties. In Fig. 7, we present the control and treatment group for 22 main sectors, and all groups of data satisfy the parallel assumption which means that DID estimation method is suitable for our model.

#### Effects of emergency response on regional electricity consumption

We first examine the impact of COVID-19 on the regional electricity consumption in easten China. The DID specification can be formulated as follows:

1 $$\begin{aligned}\ln (Y^{i,t}_{\text {regional}})&=\alpha +\beta _{0}\mathtt {Treat}^{t}\cdot \mathtt {Before}^{t}+\sum ^{4}_{n=1}\beta _{n}(\mathtt {Treat}^{t}\cdot \mathtt {After}^{t}_n) +\phi _{0}\mathtt {Before}^{t}\nonumber \\&\quad +\sum ^{4}_{n=1}\phi _{n}\mathtt {After}^{t}_n+\omega \mathtt {Treat}^t+H^{t}+B^{i}+\theta ^{t}+\epsilon ^{i,t}, \end{aligned}$$

where *i* and *t* respectively index the county and the week; the dependent varaible$\ln (Y^{i,t}_{\text {regional}})$ is the logarithm of weekly electricity consumption of counties *i* in eastern China at time *t*; $\mathtt {Treat}^{t}$ is the dummy variable of the treatment and its value is 0 when time *t* is in year 2019, and is 1 otherwise; $H^t$ is the dummy variable that accounts for holiday fixed effects, such as effects of Spring Festival holiday and Labour holiday. The county-fixed effect $B_{i}$ is included to absorb the county-specific heterogeneities that may contaminate the estimation of our interested coefficient $\beta _{n}$. We also control for the week-fixed effect $\theta ^t$ to eliminate the time-specific impact. $\epsilon ^{i,t}$ is the error term, and the standard error are clustered at the weekly level. Note that, we mainly use the weekly data for the estimation to better satisfy the parallel trend assumption. In Eq. (1), we include one pre-lockdown period indicator:$\mathtt {{Before}^{t}}$ is a dummy that takes value 1 for the period from from Jan 16 to Jan 23th in 2020 (one week before COVID-19 outbreak in eastern China), which can be used to examine the parallel trend assumption in the DID analysis; Four period indicators for the emergency response are also included in Eq. (1): $\mathtt {After}^{t}_1$ is a dummy that takes value 1 for the period from Jan 24th to Mar 1st in 2020 (the period during Level 1 emergency response), and is 0 otherwise; $\mathtt {After}^{t}_2$ is a dummy that takes value 1 for the period from Mar 2nd to Mar 22th in 2020 (the period during Level 2 emergency response), and is 0 otherwise; $\mathtt {After}^{t}_3$ is a dummy that takes value 1 for the period from Mar 23rd to Apr 30th in 2020 (the period during Level 3 emergency response), and is 0 otherwise; $\mathtt {After}^{t}_4$ is a dummy that takes value 1 for the period from May 1st to Jul 1st in 2020 (the period after Level 3 emergency response), and is 0 otherwise. The omitted benchmark period is from Jan 1st to Jan 15th, 2020. $(\mathtt {Treat}^{t}{\cdot }\mathtt {After}^{t}_i)$ is the dummy variable that equals to one at the *i*th phase in 2020, and zero otherwise.

The estimation sample used in the regressions is the 2020 data and 2019 data for weekly electricity consumption of 96 counties in eastern China. We compare the weekly electricity consumption of eastern China in 2020 to itself in the same matched lunar calendar period in 2019 without the Coronavirus outbreak. We interpret the coefficient estimate of $\mathtt {Treat}^{t}{\cdot }\mathtt {After}^{t}_1$ as measuring the causal effect of level 1 emergency response on the regional electricity consumption; the coefficient estimate of $\mathtt {Treat}^{t}{\cdot }\mathtt {After}^{t}_2$ as measuring the causal effect of level 2 emergency response on the regional electricity consumption; the coefficient estimate of $\mathtt {Treat}^{t}{\cdot }\mathtt {After}^{t}_3$ as measuring the causal effect of level 3 emergency response on the regional electricity consumption; and the coefficient estimate of $\mathtt {Treat}^{t}{\cdot }\mathtt {After}^{t}_4$ as measuring the post-pandemic recovery of the regional electricity consumption after stopping level 3 emergency response; The coefficient estimate of $\mathtt {Treat}^{t}{\cdot }\mathtt {Before}^{t}$ examines whether the parallel trend assumption for DID is satisfied. The possibly time-varying Spring Festival effects and the holiday effects are both absorbed in the week-fixed effects and holiday-fixed effect.

#### Effects of emergency response on industry’s electricity consumption

In order to examine how response to COVID-19 varies with industries, we use the DID specification to examine the impact of different control measures on the electricity consumption of the specific industry, which can be described as

2 $$\begin{aligned}\ln (Y^{i,t}_{\text {sector}})&=\alpha +\beta _{0}\mathtt {Treat}^{t}\cdot \mathtt {Before}^{t}+\sum ^{4}_{n=1}\beta _{n}(\mathtt {Treat}^{t}\cdot \mathtt {After}^{t}_n) +\phi _{0}\mathtt {Before}^{t}\nonumber \\&\quad +\sum ^{4}_{n=1}\phi _{n}\mathtt {After}^{t}_n+\omega \mathtt {Treat}^t+H^{t}+B^{i}+\theta ^{t}+\epsilon ^{i,t}, \end{aligned}$$

where $\ln (Y^{s,t}_{\text {sector}})$ represents for the logarithm of weekly electricity consumption of a specific industry in county *i* at week *t*; $H^t$ is the dummy variable that accounts for holiday fixed effects, such as effects of Spring Festival holiday and Labour holiday. $B^i$ is the county variable that accounts for county fixed effects, and $\theta ^t$ is the week-fixed effect; $\mathtt {Treat}^{t}$ is the dummy variable of the treatment and its value is 0 when time t is in year 2019, and is 1 otherwise. $\mathtt {Before}^{t}$,$\mathtt {After}^{t}_1$,$\mathtt {After}^{t}_2$,$\mathtt {After}^{t}_3$, $\mathtt {After}^{t}_4$ are defined in the same way as in Eq. (1). The omitted benchmark period is from Jan 1st to Jan 15th, 2020. $\epsilon ^{i,t}$ is the error term, and the standard error are clustered at the weekly level. The estimated coefficients, $\beta _1$, $\beta _2$, $\beta _3$, and $\beta _4$, respectively represent the casual impacts of industy’s emergency response on electricity consumption in *n*th phase. $\beta _0$ is used to check the parallel trend assumption.

The estimation sample used in the regressions is the 2020 data and 2019 data for weekly electricity consumption of 53 sub-sectors in 96 counties in eastern China. For each sub-sector, the emprical analysis is conducted based on Eq. (1) to estimate the impacts of emergency response and industry’s economic recovery.

#### Lag effects of COVID-19 outbreak on industry’s electricity consumption

Finally, to estimate the industry’s resilience to COVID-19, we design the empirical analysis to figure out the lag effect of the COVID-19 outbreak on industries from Jan 24th, 2020. We describe the estimation model of dynamic lag effect of COVID-19 on 53 sub-sectors as

3 $$\begin{aligned}\ln (Y^{i,t}_{\text {sector}})&=\alpha +\beta _{0}(\mathtt {Treat}^{t}{\cdot }\mathtt {T_1})+\sum ^{24}_{n=2}\beta _{n}(\mathtt {Treat}^{t}{\cdot }\mathtt {T}_n) +\phi _{1}\mathtt {T}_1\nonumber \\&\quad +\sum ^{24}_{n=2}\phi _{n}\mathtt {T}_n+\omega \mathtt {Treat}^t+B^{i}+H^{t}+\theta ^{t}+\epsilon ^{i,c,t}, \end{aligned}$$

where dependent variable, $\ln (Y^{s,t}_{\text {sector}})$, represents for the logarithm of weekly electricity consumption of a specific industry in county *i* at week *t*. $\mathtt {Treat}^{t}$ is the dummy variable of the treatment and its value is 0 when time t is in year 2019, and is 1 otherwise. Our data records the electricty consumption of industries covering 1 week before the COVID-19 outbreak (from Jan 16th to Jan 23rd in 2020) and 23 weeks after the outbreak (from from Jan 24th to Jul 1st in 2020). Thus, in Eq. (3) there are 24 period indicators in equation (3) and $\mathtt {T_i}$ is a dummy that takes value 1 for the period of week *i*. Among all, week 2 is the Spring Festival Holiday meanwhile the COVID-19 outbreak. The estimated coefficient of $\beta _i$ represents the lag effects of the COVID-19 outbreak on electricity consumption in ${i^{th}}$ week. $\beta _0$ is used to check the parallel trend assumption. $B_{i}$,$H^t$ and $\theta ^t$ are The county-fixed effect, holiday-fixed effect and week-fixed effect to absorb the county-specific heterogeneities that may contaminate the estimation of our interested coefficient $\beta _{n}$. $\epsilon ^{i,t}$ is the error term, and the standard error are clustered at the weekly level.
